# Adaptive and lightweight surrogate gradients: enhancing training efficiency of spiking neural networks

**DOI:** 10.3389/fnins.2026.1795946

**Published:** 2026-03-13

**Authors:** Kungjui Hou, Kunlun Wu, Yongcheng Zhou

**Affiliations:** 1 Lingyange Semiconductor Inc., Zhuhai, Guangdong, China; 2 School of Artificial Intelligence, Anhui University, Hefei, China; 3 School of Automation, Chongqing University, Chongqing, China

**Keywords:** adaptive training, lightweight backpropagation, spiking neural networks, surrogate gradient, training efficiency

## Abstract

**Background:**

Spiking Neural Networks (SNNs) have emerged as a promising paradigm in artificial intelligence due to their energy efficiency. However, training SNNs remains a formidable challenge because the nondifferentiable nature of spike activation functions prevents the direct application of conventional backpropagation. Existing surrogate gradient methods often suffer from critical limitations, including gradient mismatch, gradient explosion or vanishing, and high computational overhead.

**Methods:**

In this paper, we propose an Adaptive and Lightweight (AdaLi) backpropagation method to address these issues. AdaLi reduces the computational complexity of the training process by introducing lightweight surrogate gradients and dynamically adjusting gradient update boundaries. Furthermore, it employs an adaptive mechanism to adjust the surrogate gradients based on training epochs, thereby enhancing network stability. The method also provides additional hyperparameters to address gradient mismatch, which can be either manually fine-tuned or automatically determined based on the distribution of spiking neuron membrane potentials.

**Results:**

Experimental results on both static and neuromorphic datasets demonstrate that SNNs trained with AdaLi outperform their baseline counterparts in terms of efficiency and accuracy. The stable surrogate gradients in AdaLi effectively mitigate the issues of gradient vanishing and explosion.

**Conclusion:**

The AdaLi method introduces a novel approach to optimizing gradient calculation and parameter updates in SNNs, paving the way for more effective and accurate training. The source code is available at https://github.com/parania/AdaLi.

## 1 Introduction

The rapid advancement of artificial neural networks (ANNs) has revolutionized various domains of machine intelligence (Wang et al., 2023; Liu et al., 2023; He et al., 2016; Ronneberger et al., 2015; Bewley et al., 2016). However, ANNs rely on dense-valued tensors for computation, a mechanism fundamentally distinct from the brain's sparse, event-driven processing of information (Guo et al., 2024; Shen et al., 2024).

This discrepancy underscores the challenge of replicating the brain's efficiency and adaptability in artificial systems. Spiking neural networks (SNNs) have emerged as a promising alternative, offering higher biological fidelity by mimicking the use of sparse, binary spike events for communication and computation (Mainen and Sejnowski, 1995; Zhang et al., 2022b, 2023). By leveraging sparsity and asynchrony (Zhang et al., 2024; Histed et al., 2009), SNNs enable energy-efficient, event-driven processing that aligns more closely with the natural mechanisms (Yamazaki et al., 2022; Zhang et al., 2022a; Zhou et al., 2024). Moreover, SNNs excel at encoding information through spatio-temporal dynamics (Baronig et al., 2025), making them particularly suited for tasks involving time-series data or neuromorphic hardware (Painkras et al., 2013; Zhang et al., 2023; Luo et al., 2023; Merolla et al., 2014; Davies et al., 2018; Pei et al., 2019; Ye et al., 2023; Liu et al., 2024; Zhang and Zhang, 2025).

Despite their potential, training SNNs remains a significant challenge due to the non-differentiable nature of the spiking activation function (Guo et al., 2023b; Eshraghian et al., 2023; Rançon et al., 2022). This non-differentiability prevents the direct application of backpropagation, a cornerstone of ANN training, limiting the optimization methods available for SNNs. While surrogate gradient methods have been proposed to address this issue (Deng et al., 2022; Neftci et al., 2019; Meng et al., 2022; Guo et al., 2022a; Wu et al., 2018; Meng et al., 2023), they often introduce gradient mismatch problems (Zenke and Vogels, 2021), leading to performance degradation during training (Guo et al., 2022b). Additionally, the spike activation function's infinite derivative at the threshold and zero derivative elsewhere exacerbate issues of gradient vanishing and explosion, further destabilizing the training process (Liang et al., 2023; Yao et al., 2023; Lian et al., 2024).

To this end, this paper proposes an Adaptive and Lightweight (AdaLi) backpropagation method to optimize SNN training. AdaLi introduces a lightweight surrogate gradient that reduces computational complexity while maintaining stability, addressing the gradient vanishing and explosion issues inherent in traditional surrogate gradients. Furthermore, AdaLi incorporates an adaptive mechanism that dynamically adjusts gradient update boundaries based on training epochs, enhancing training stability and efficiency. This adaptive function also provides hyperparameters to mitigate gradient mismatch, which can be manually fine-tuned or automatically determined based on the distribution of spiking neuron membrane potentials. By these design, AdaLi optimizes the network training process, achieving satisfactory results across various networks and datasets. A comparison of the properties between the AdaLi method and other methods is illustrated in Table 1.

**Table 1:** Comparison of the ANN-to-SNN conversion, traditional surrogate gradient (SG), and AdaLi method with respect to latency, compute density, performance with low latency, and applicability on neuromorphic data.

**Metric**	**Conversion**	**SG**	**AdaLi**
Latency	High	Low	Low
Compute density	High	Medium	Low
Performance w/low latency	Low	Medium	High
Neuromorphic data	Non-applicable	Applicable	Applicable

The main contributions of this work can be summarized as follows:

- We designed a lightweight surrogate gradient that reduces computational complexity during backpropagation, making it particularly effective for training large-scale networks. This design inherently resolves the gradient vanishing and explosion issues prevalent in traditional surrogate gradients.
- We proposed an adaptive function that autonomously controls the range of gradient updates, maximizing training stability while reducing the computational overhead. This function also provides hyperparameters to mitigate gradient mismatch, which can be flexibly adjusted or automatically set based on neuron membrane potential distributions.
- We evaluated AdaLi on both static and neuromorphic datasets, demonstrating its superior performance across various network architectures and tasks. Experimental results confirmed that AdaLi achieves high efficiency and accuracy, outperforming other SOTA methods.

## 2 Related work

In this section, we provide a brief overview of the network learning methods employed for SNNs, with a particular focus on direct training approaches.

### 2.1 Network learning of SNNs

The training of SNNs typically involves three primary methodologies: ANN-to-SNN conversion, unsupervised learning paradigms [e.g., Spike-Timing-Dependent Plasticity (STDP) (Caporale and Dan, 2008; Dan and Poo, 2004; Rahman and Yusoff, 2025)], and direct training using surrogate gradients. ANN-to-SNN conversion approximates the firing dynamics of an SNN by utilizing the activation patterns of an ANN with the similar architecture and weights configuration (Rueckauer et al., 2017). This method directly derives SNN parameters from a pre-trained ANN, ideally ensuring comparable performance to its ANN counterpart, with minimal performance degradation. However, this approach often incurs increased latency to replicate the high-resolution activation values of the ANN, thereby compromising the energy efficiency that is a core advantage of SNNs.

STDP, a prominent unsupervised learning method for SNNs, modulates synaptic efficacy based on the precise timing of pre- and post-synaptic spikes (Brzosko et al., 2019; Saponati and Vinck, 2023; Debanne and Inglebert, 2023). While STDP has significant potential in shaping network connectivity and information processing, its inability to capture global information poses challenges for convergence in large-scale models, particularly with complex datasets (Masquelier and Thorpe, 2007; Tavanaei and Maida, 2016; Diehl and Cook, 2015).

In contrast to aforementioned methods, direct training approaches based on surrogate gradients offer notable advantages, particularly in reducing inference latency, which improves computational efficiency while preserving the energy efficiency inherent to SNNs (Wu et al., 2019; Su et al., 2023; Zhou et al., 2024). Moreover, surrogate gradient-based training methods address the challenges associated with unsupervised learning, especially for large-scale datasets. By incorporating established gradient-based optimization algorithms, these techniques have demonstrated robust performance across a wide range of datasets.

### 2.2 Surrogate gradients based direct training

Direct training methods can be broadly categorized into time-based and activation-based approaches, depending on whether the gradient reflects spike timing (time-based) (Cai et al., 2023) or spike magnitude (activation-based) (Guo et al., 2023b; Zhu et al., 2022).

Time-based methods adjust spike timing by directing whether to advance or delay spike emission. Works such as (Bohte et al. 2002) and (Hong et al. 2019) distribute errors across each firing time rather than through individual neurons, using the negative reciprocal of the time derivative of the membrane potential to estimate spike timing derivatives. Despite reducing computational complexity, training deep time-based SNN models on large-scale datasets remains challenging (Mostafa, 2018; Xiao et al., 2022).

Activation-based methods (Fang et al., 2021a; Rathi and Roy, 2020) use differentiable surrogate functions to approximate the non-differentiable spike activity function for gradient calculation during backpropagation. (Zenke and Ganguli 2018) introduced SuperSpike, a supervised learning method for training multilayer SNNs using non-zero surrogate gradients to address gradient-related issues. (Cheng et al. 2020) encoded visual features into spike sequences and trained SNN models with backpropagation, incorporating lateral interactions. However, fixed surrogate gradient forms in these methods make it difficult to address the issue of gradient mismatch. (Li et al. 2021) proposed differentiable spike functions based on hyperbolic tangent functions, incorporating finite difference gradients and adjusting a temperature factor to modify the function's shape. (Guo et al. 2022a) introduced evolutionary surrogate gradients, using smaller coefficients during early training stages to improve convergence and accuracy. Despite their dynamic adjustments, these methods' complexity and numerous parameters increase computational demands, making them less suitable for large-scale networks and prone to gradient vanishing and explosion issues.

In contrast, the surrogate gradients in our proposed AdaLi method are computationally efficient and mitigate gradient vanishing and explosion issues. AdaLi adjusts parameters based on the distribution of neuron membrane potentials, improving gradient mismatch problems. Combined with an adaptive function, AdaLi reduces the gradient computation overhead during training while enhancing SNN training stability.

## 3 Proposed method

### 3.1 Framework overview

**Figure 1:**
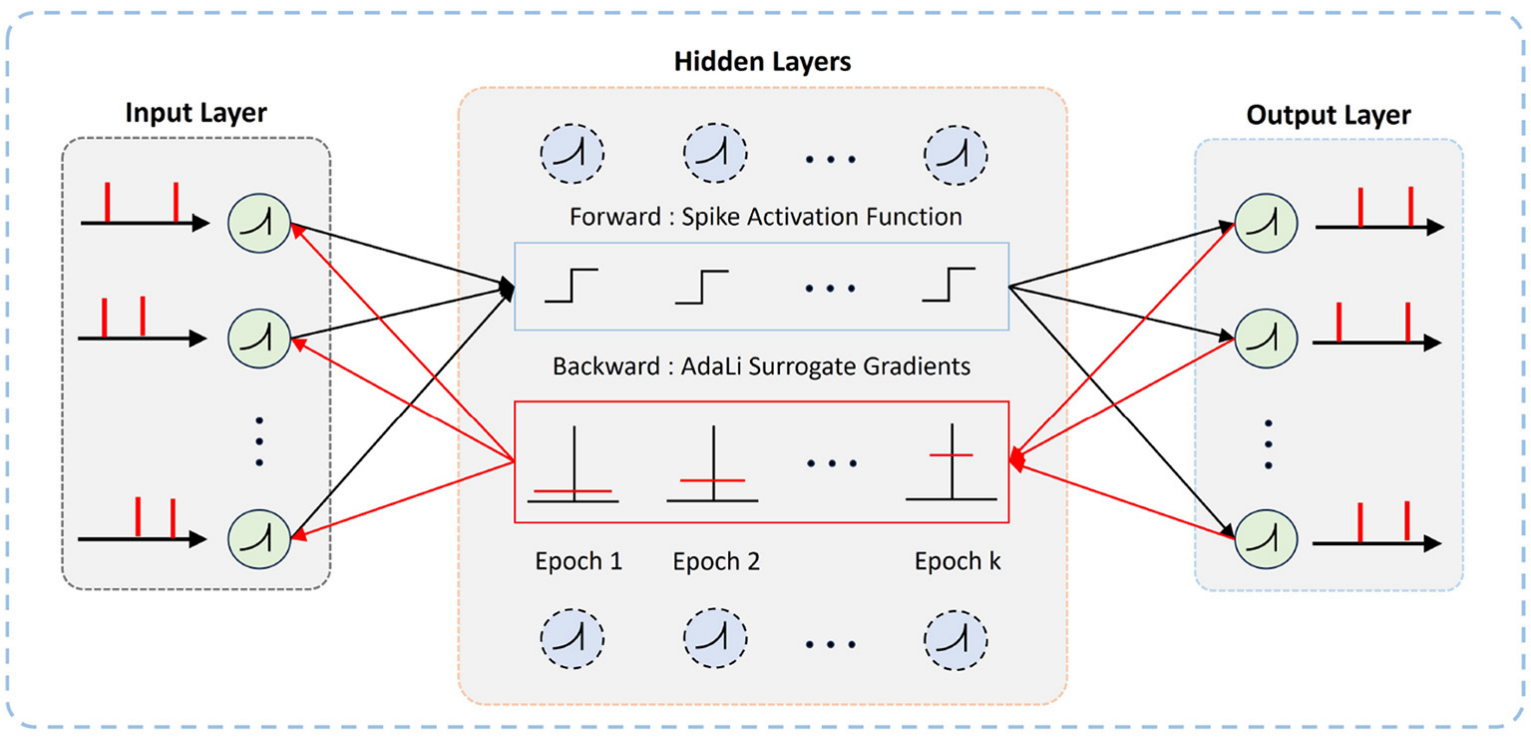
The overall framework of our proposed AdaLi method. The method automatically evolves in each epoch in backpropagation to reduce the range of parameters update.

**Figure 2:**
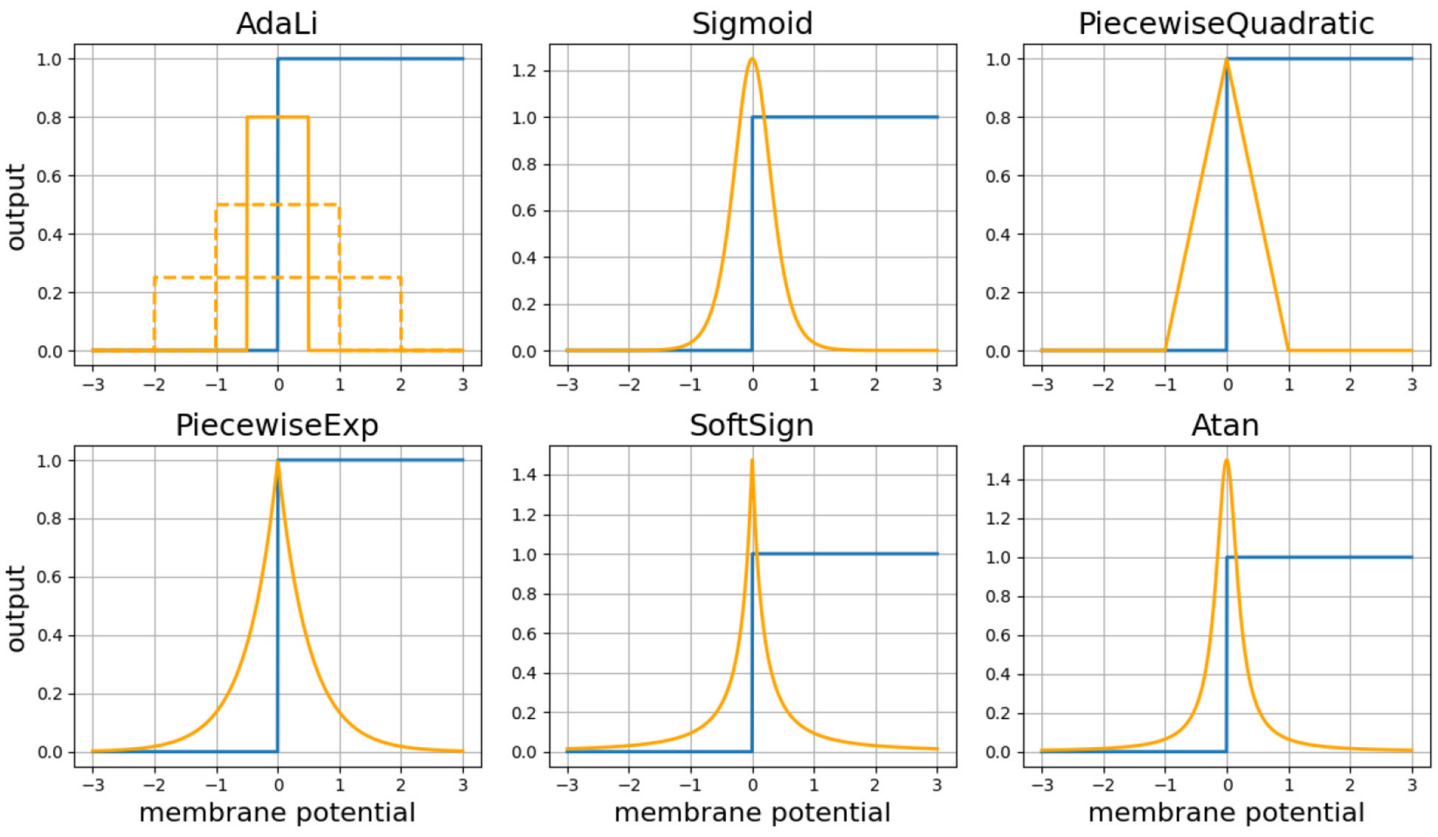
The illustration delineates six distinct activation functions along with their corresponding surrogate gradient curves. The blue curve corresponds to the forward propagation process, whereas the orange curve depicts the surrogate gradient utilized during backpropagation. The dashed lines in the AdaLi activation function subplot represent the surrogate gradients across different epochs, which gradually narrow inward with training progression and shrink the effective gradient update range. This contraction trend is positively correlated with the training effect, balancing the stability of early training and the computational efficiency of later training stages.

Figure 1 illustrates the comprehensive diagram of our proposed AdaLi method. In the forward propagation phase, spiking neurons utilize the Heaviside function to determine spike emission. During backpropagation, parameters are no longer updated by calculating the gradient of the Heaviside function. Instead, AdaLi introduces surrogate gradients that adapt over the course of training epochs, thereby enhancing network performance while concurrently reducing computational demands. As shown in Figure 2, a comparison in shape between our proposed AdaLi and other commonly surrogate gradients-based methods.

Additionally, the representation of information in SNNs differs from that of ANNs. SNNs leverage spike sequence transmissions, primarily employing two encoding strategies: temporal coding and rate coding. Temporal coding emphasizes precise spike timing, whereas rate coding focuses on spike frequency as the conduit of information. Temporal coding is typically recognized for its energy-efficient deployment on neuromorphic hardware due to the emission of sparse spikes. However, temporal coding may necessitate configurations less conducive to chip compatibility or exhibit optimal performance only with simplistic datasets, as noted in (Meng et al. 2022). In this paper, we employ rate coding as our methodology for SNN training due to its robustness.

**Figure 3:**
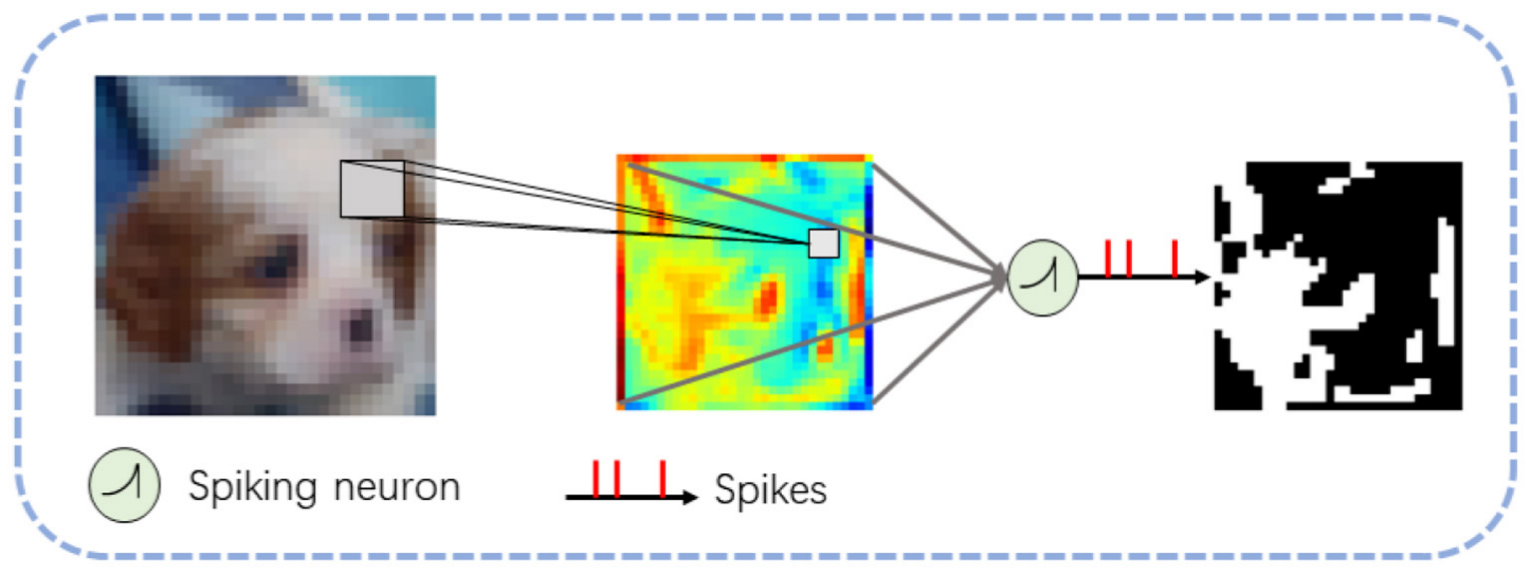
Representation of images in SNNs, the first layer of the network encodes the image from continuous values to discrete spike signals.

Considering that the pixel values of static datasets are continuous, while SNNs typically receive discrete spike signals as input and output discrete spike signals, we designate the first convolutional layer and spiking neurons of the network as encoding units for the input, as shown in Figure 3. After the convolutional layer processes the input image to generate feature maps, the first spiking neuron receives these feature maps as input, encodes them into discrete spike signals, and transmits them to the next layer.

Assuming a two-dimensional input data *X*, the operation of encoding input data into spikes can be given as:


$$\begin{array}{l} \text{Encoder}\left(X\right)=S\left(\text{Conv}\left(X\right)\right) \end{array}$$ (1)

where Conv denotes the convolution operation, *S* denotes the spiking neurons' firing operation, and the firing process of spiking neurons will be elaborated later in Section 3.2. Let the convolution kernel (filter) be *W*, the convolution operation is expressed as:


$$\begin{array}{c} Y\left(i,j\right)=\left(X*W\right)\left(i,j\right)\\ =\sum_{m}\sum_{n}X\left(m,n\right)\cdot W\left(i-m,j-n\right) \end{array}$$ (2)

where *Y*(*i, j*) is the element of the output feature map with *i* and *j* being the row and column indices. *m* and *n* denote the row and column indices of the input feature map. More specifically, the encoding operation can be represented as:


$$\begin{array}{l} \text{Encoder}\left(X\right)=H\left(W*X-V_{th}\right) \end{array}$$ (3)

where *V*_*th*_ denotes the firing threshold of spiking neurons, and *H* is the Heaviside function defined as:


$$H(x)=\{\begin{array}{ll} 1, & x\geq 0\\ 0, & x<0 \end{array}$$ (4)

### 3.2 Forward pass

Inspired by the communication mechanisms in biological neurons, spiking neurons emulate these intricate interactions using binary signals. This paper leverages the widely adopted integrate-and-fire (IF) and leaky-integrate-and-fire (LIF) models (Burkitt, 2006) to streamline the simulation of spike generation and subsequent binary signal communication among neurons. Upon receiving a spike signal, it is integrated into the neuron's membrane potential, akin to electrical charge accumulation. This accumulation process, which captures the dynamic behavior of the membrane potential, can be expressed mathematically as follows:


$$\begin{array}{lll} \text{IF:} & \;\frac{\text{d}V\left(t\right)}{\text{d}t}=I\left(t\right), & V<V_{th} \end{array}$$ (5)

$$\begin{array}{lll} \text{LIF:} & \;\tau \frac{\text{d}V\left(t\right)}{\text{d}t}=-\left(V\left(t\right)-V_{rest}\right)+I\left(t\right), & V<V_{th} \end{array}$$ (6)

where τ is the membrane time constant, *V*_*rest*_ is the resting potential, and *I* is the input current linked to the received spikes. When the membrane potential *V* exceeds the predefined threshold *V*_*th*_ at time *t*_*f*_, the neuron initiates a spike and resets its membrane potential to *V*_*rest*_. The output spike train can be formulated using the Dirac delta function $s\left(t\right)=\underset{t_{f}}{\sum }\delta \left(t-t_{f}\right)$.

The aforementioned scenario depicts the continuous process of neuron dynamics. As computers can only approximate continuity through discrete representations, it is essential to understand the discrete representation of neuron dynamics. The model is represented in discrete form as:


$$\begin{array}{l} U\left[n\right]=f\left(V\left[n-1\right],I\left[n\right]\right) \end{array}$$ (7a)

$$\begin{array}{l} s\left[n\right]=H\left(U\left[n\right]-V_{th}\right) \end{array}$$ (7b)

$$\begin{array}{l} V\left[n\right]=U\left[n\right]-V_{th}s\left[n\right] \end{array}$$ (7c)

where the variables *U*[*n*], *s*[*n*], and *V*[*n*] indicate the accumulated membrane potential upon receiving input, the output spikes, and the membrane potential after spike emission, respectively. If a spike is emitted, *V*[*n*] represents the membrane potential post-emit, whereas if no spike is emitted, *V*[*n*] equals *U*[*n*]. *H*(*x*) represents the Heaviside step function, signifying the process of spike generation. The function *f*(·, ·) denotes the membrane potential update function, taking the previous membrane potential and current input currents as inputs, and is defined as:


$$\begin{array}{ll} \text{IF:}\; & f\left(V,I\right)=V+I \end{array}$$ (8)

$$\begin{array}{ll} \text{LIF:}\; & f\left(V,I\right)=e^{-\frac{\text{Δ}t}{\tau }}V+\left(1-e^{-\frac{\text{Δ}t}{\tau }}\right)I \end{array}$$ (9)

In the LIF model, Δ*t* < τ represents the discrete step, typically set to be significantly smaller than τ in practice. By combining Equations 7a, c, we derive a more condensed update rule for the membrane potential:


$$\begin{array}{l} V\left[n\right]=f\left(V\left[n-1\right],I\left[n\right]\right)-V_{th}s\left[n\right] \end{array}$$ (10)

Equation 10 is employed to establish the forward pass of SNNs. Assuming the SNN comprises *L* layers and follows a feedforward structure, the dynamic process of neurons in the IF model can be represented by integrating Equations 8, 10 as:


$$\begin{array}{l} {\text{V}}^{i}\left[n\right]={\text{V}}^{i}\left[n-1\right]+V_{th}^{i-1}{\text{W}}^{i}{\text{s}}^{i-1}\left[n\right]-V_{th}^{i}{\text{s}}^{i}\left[n\right] \end{array}$$ (11)

where the index *i* = 1, 2, ⋯ , *L* iterates through the layers, with *s*^*i*^ representing the output spike of the *i*^th^ layer, generated by Equation 7b. **W**^*i*^ denotes the weight parameters from the (*i*−1)^th^ layer to the *i*^th^ layer. $V_{th}^{i-1}{\text{W}}^{i}{\text{s}}^{i-1}\left[n\right]$ is the input currents for the *i*^th^ layer. The spike thresholds are uniform across all layers. Similarly, by combining Equations 9, 10, the dynamic of neurons in the LIF model can be represented as:


$$\begin{array}{c} {\text{V}}^{i}\left[n\right]=\exp\left(-\frac{\text{Δ}t}{\tau^{i}}\right){\text{V}}^{i}\left[n-1\right]\\ +(1-\exp\left(-\frac{\text{Δ}t}{\tau^{i}}\right))\text{Δ}tV_{th}^{i-1}{\text{W}}^{i}{\text{s}}^{i-1}\left[n\right]-V_{th}^{i}{\text{s}}^{i}\left[n\right] \end{array}$$ (12)

### 3.3 Proposed AdaLi method

The spike activation function in Equation 7b can be considered a variant of the sign function. Its derivative is zero when the membrane potential is not equal to the threshold potential, and infinite when the membrane potential equals the threshold potential. Due to this non-differentiable characteristic, the backpropagation algorithm cannot be directly applied to SNNs. For a more detailed demonstration, we provide the expression for calculating parameter gradients:


$$\frac{\partial L}{\partial W_{l}}=\sum_{t}\left(\frac{\partial L}{\partial O_{l}^{t}}\frac{\partial O_{l}^{t}}{\partial U_{l}^{t}}+\frac{\partial L}{\partial U_{l}^{t+1}}\frac{\partial U_{l}^{t+1}}{\partial U_{l}^{t}}\right)\frac{\partial U_{l}^{t}}{\partial W_{l}}$$ (13)

where L is the loss function, **W**_*l*_ is the weight matrix of layer *l*., and $O_{l}^{t}$ represents the output of neuron in layer *l* at time *t*. The gradient of the spike activation function at time step *t* in the *l*_*th*_ layer is denoted by $\frac{\partial O_{l}^{t}}{\partial U_{l}^{t}}$. The weight update rules are as follows:


$$\begin{array}{l} \text{W}=\text{W}-\eta \text{Δ}\text{W} \end{array}$$ (14)

where η denotes the update rate and Δ**W** denotes the weight update amount. In Equation 7b, the neuron's firing process is described by a Heaviside step function, making its derivative the Dirac delta function:


$$\delta (U)=\{\begin{array}{ll} +\infty , & U=0\\ 0, & U\neq 0 \end{array}$$ (15)

where *U* represents the membrane potential of a spiking neuron subtracted by the threshold before the neuron fires a spike.

Directly employing the Dirac delta function for gradient descent introduces substantial instability into the network's training regimen. The predominant zero-value scenario results in zero changes to the weight adjustments, Δ**W**, rendering network parameter updates ineffectual. Conversely, in scenarios where the derivative encounters infinite values, Δ**W** escalates to infinity, leading to exceedingly erratic and unpredictable updates of the network parameters.

It is widely acknowledged that gradients indicate the direction and rate of change of a function at a given point. However, due to the discontinuity of the spike activation function at the threshold, gradients are zero everywhere except at the threshold. In fact, the points with gradient equal to zero also have a trend of numerical change at the macro level. Therefore, the gradient of the spike activation function is unable to provide suitable direction or the rate of change. This paper proposes that this is why traditional gradient descent algorithms are challenging to apply to SNNs. To successfully apply gradient descent algorithms to SNNs, the key is not merely handling the gradient at the threshold potential, but reconstructing the direction and rate of change of the spike activation function for most membrane potential values.

The discontinuity point of the spike activation function is at the threshold potential. Determining the rough direction of change of the spike activation function at a given point is straightforward. When the membrane potential is less than the threshold, an increase in membrane potential may cause the spike activation function value to increase from 0 to 1. Conversely, when the membrane potential is greater than the threshold potential, a decrease in membrane potential may cause the spike activation function value to decrease from 1 to 0. Once the direction of change is determined, further research on the rate of change of the spike activation function at a given point is conducted. An intuitive approach is to determine the rate of change of the spike activation function at a given point based on the range of membrane potential that needs gradient updates. If there is no restriction on the range of membrane potential that needs gradient updates, outliers in the membrane potential may lead to unstable training when updating their gradients.

Suppose V^−^ and V^+^ are the boundaries for the gradient update of membrane potential, generally, ${\text{V}}^{-}<{\text{V}}_{th}$ and ${\text{V}}^{+}>{\text{V}}_{th}$, we can obtain the basic form of the surrogate gradient:


$$\phi (U)=\{\begin{array}{l} \frac{\alpha }{V_{th}-V^{-}},U<V_{th}\\ \frac{\beta }{V^{+}-V_{th}},U\geq V_{th} \end{array}$$ (16)

where $\frac{\alpha }{V_{th}-V^{-}}$ and $\frac{\beta }{V^{+}-V_{th}}$ are the reconstructed rate and direction of change of the spike activation function on the left and right sides of the threshold, respectively. *U* represents the membrane potential of the spiking neuron, while α and β are appropriate constant gradient intensity factors obtained experimentally to mitigate the gradient mismatch issue effectively. The selection of α and β is discussed in Section 4.2.

Compared to the traditional methods of using the sigmoid function as a surrogate for the spike activation function, our approach simplifies the calculation of membrane potential gradients during backpropagation, making it a lightweight backpropagation method. As the form of surrogate gradients is always constant, stable values prevent gradient vanishing and exploding issues.

The training process encounters significant challenges due to the overly narrow update range, leading to a dearth of gradient updates during backpropagation, and conversely, an excessively expansive update range that accommodates outliers, triggering instability and amplifying the computational burden. To address these issues, we craft adaptive functions designed to gradually constrict the gradient update range at distinct rates as training epochs progress. This novel approach mitigates the computational intensity associated with backpropagation. For a logarithmic reduction rate for the gradient update range, we define the adaptive function as follows:


$$\begin{array}{l} V_{k}=V_{1}\cdot {\frac{V_{N}}{V_{1}}}^{\left(\log k/\log N\right)} \end{array}$$ (17)

Thus, to decrease the update range of gradients with a linear rate of change, the expression for the adaptive function is:


$$\begin{array}{l} V_{k}=\frac{V_{N}-V_{1}}{N-1}\cdot \left(k-1\right)+V_{1} \end{array}$$ (18)

where *V*_*k*_ has two forms, $V_{k}^{-}$ and $V_{k}^{+}$, representing the boundaries of membrane potential requiring gradient updates on the left and right sides of the threshold in the *k*-th training round. *N* and *k* denote the total epochs and the current epoch, respectively. The values of $V_{1}^{-}$ and $V_{1}^{+}$ are the initial boundaries of membrane potential that needs gradient updates. We choose a relatively large value at the start of training to stabilize the training process. $V_{N}^{-}$ and $V_{N}^{+}$ are the boundaries of membrane potential requiring gradient updates at the end of training. Their calculation formulas are as follows:


$$\begin{array}{l} V_{N}^{\pm }=V_{th}\pm p\cdot |V_{th}-V_{1}^{\pm }| \end{array}$$ (19)

where p is a constant between 0 and 1. This formula implies that the final update boundary is set to a fixed proportion p of the distance from the threshold *V*_*th*_ to the initial boundary $V_{1}^{\pm }$. The selection of hyperparameters is discussed in Section 4.2. For clearer illustration, we present the pseudocode of one iteration of SNN training with our AdaLi method in Algorithm 1.

Algorithm 1 **One iteration of SNN training with the proposed AdaLi method.**

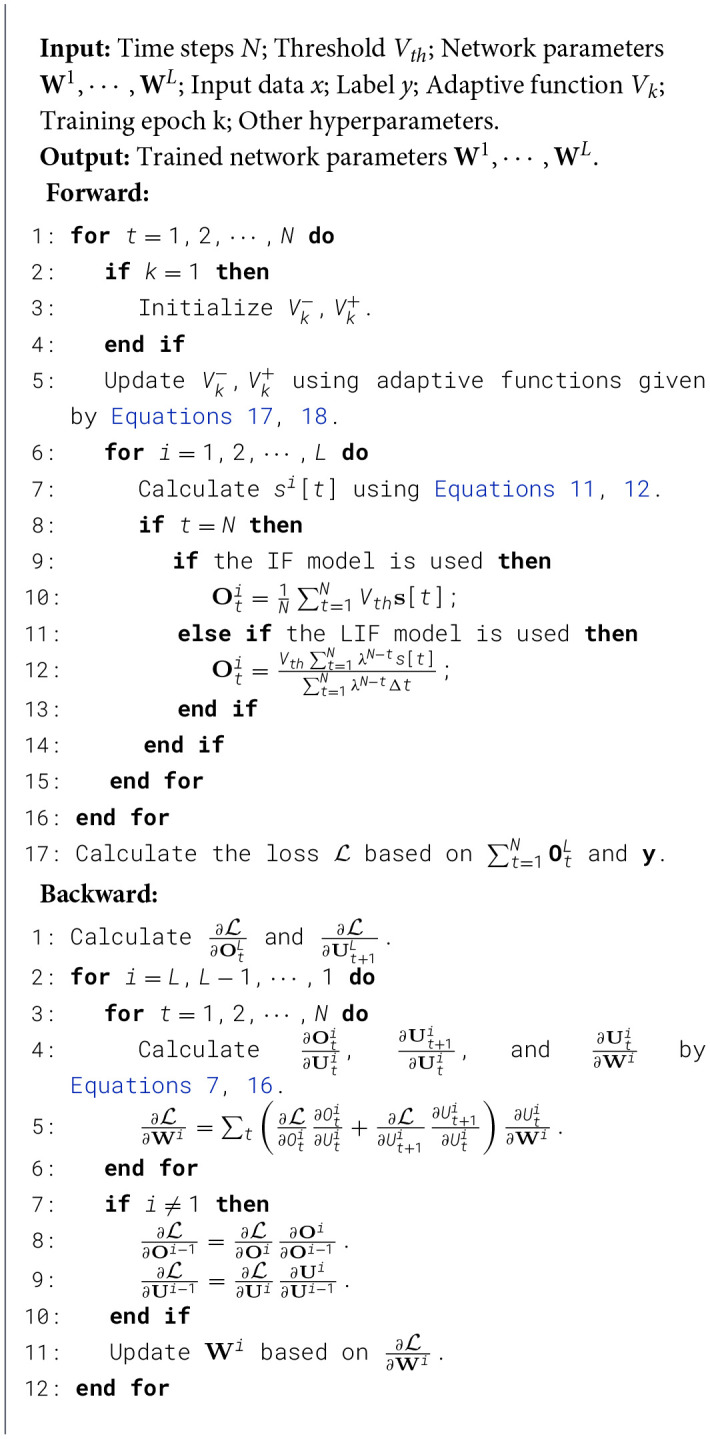

During the initial training phase, a substantial gradient update scope, encompassing upwards of 50% of the membrane potential gradient values, is established to secure the training process's stability. As training progresses and stability becomes assured, the gradient update extent is reduced, diminishing the computational overhead associated with the backpropagation gradients. Upon training completion, the gradient update bandwidth is minimized, optimizing performance efficiency.

## 4 Experimental results

In this section, we demonstrate the effectiveness of our proposed AdaLi method through a comprehensive set of experimental results. We conducted experiments using established neural network architectures, including ResNet18 (He et al., 2016), VGG models (VGG11 and VGG16) (Simonyan and Zisserman, 2014), and evaluated them on widely-used static image benchmark datasets, such as CIFAR-10 and CIFAR-100 (Krizhevsky and Hinton, 2009). Further, to showcase the adaptability of AdaLi to spatiotemporal visual data, we extended our evaluation to neuromorphic datasets, specifically CIFAR-10-DVS (Li et al., 2017) and DVS128Gesture (Amir et al., 2017).

### 4.1 Comparisons with other methods

We compared our AdaLi with other SOTA methods on both static and neuromorphic datasets, as shown in Tables 2, 3. On the CIFAR-10 dataset, our method achieved a top-1 accuracy of 95.33% using ResNet18 with 4 time steps, surpassing the performance of (Deng et al. 2022) by 0.89% under the same conditions. Notably, even with a single time step, AdaLi achieved a top-1 accuracy of 94.65% using ResNet18, outperforming the results obtained with 6 time steps in (Zheng et al. 2021). For CIFAR-100, AdaLi reached a top-1 accuracy of 76.22% using ResNet20 with 4 time steps, which is 3.92% higher than the performance reported in (Guo et al. 2023c) with the same network architecture and time steps. Additionally, our method outperformed results achieved with 10 time steps in (Guo et al. 2023a) using just 4 time steps with the VGG16 network architecture. These experimental results demonstrated that the AdaLi method delivers low latency and high performance on static datasets.

**Table 2:** Performance comparison of the AdaLi with other approaches on the static datasets.

**Dataset**	**Method**	**Type**	**Architecture**	**Neuron model**	**Timestep**	**Accuracy**
CIFAR-10	SATP (Zhang et al., 2024)	ANN-to-SNN conversion	VGG16	RMP-IF	128	91.92%
	Event-driven BP (Zhu et al., 2022)	Direct training	ResNet14	LIF	\	92.45%
	TSSL-BP (Zhang and Li, 2020)	Direct training	CIFARNet	LIF	5	91.41%
	FT-SNN (Stanojevic et al., 2024)	Direct training	\	LIF	\	93.69%
	MPD-ATP (Shan et al., 2025)	Direct training	\	LIF	8	94.27%
	STD-ED (Wei et al., 2024)	Direct training	\	LIF	\	94.33%
	HP-SNN (Wu et al., 2022)	Hybrid training	\	LIF	4.5 (average)	91.08%
	PLIF (Fang et al., 2021b)	Direct training	PLIFNet	PLIF	8	93.50%
	Dspike (Li et al., 2021)	Direct training	ResNet18	LIF	6	94.25%
	DSR (Meng et al., 2022)	Direct training	ResNet18	LIF	20	95.40%
	BKDSNN (Xu Z. et al., 2024)	Direct training	ResNet18	IF	4	93.41%
	TET (Deng et al., 2022)	Direct training	ResNet18	LIF	2	94.16%
					4	94.44%
	STBP-tdBN (Zheng et al., 2021)	Direct training	ResNet18	Iterative LIF	6	93.16%
					4	92.92%
					2	92.34%
	IM-Loss (Guo et al., 2022a)	Direct training	ResNet18	LIF	2	93.85%
			VGG16		5	93.85%
			CIFARNet		4	92.20%
	RecDis-SNN (Guo et al., 2022b)	Direct training	ResNet18	LIF	2	93.64%
					4	95.53%
	Real Spike (Guo et al., 2022c)	Direct training	ResNet18	LIF	2	95.31%
					4	95.51%
			ResNet20		4	91.89%
	**AdaLi** (ours)	Direct training	ResNet18	LIF	1	**94.65%**±0.05
					2	**95.03%**±0.07
					4	**95.33%**±0.03
			VGG16	LIF	4	**94.74%**±0.04
CIFAR-100	Event-driven BP (Zhu et al., 2022)	Direct training	VGG11	LIF	\	63.97%
	FT-SNN (Stanojevic et al., 2024)	Direct training	\	LIF	\	72.24%
	MPD-ATP (Shan et al., 2025)	Direct training	\	LIF	8	74.41%
	STD-ED (Wei et al., 2024)	Direct training	\	LIF	\	73.01%
	LTL (Yang et al., 2022)	Tandem learning	ResNet20	LIF	31	76.08%
	RecDis-SNN (Guo et al., 2022b)	Direct training	ResNet18	LIF	4	74.01%
	IM-Loss (Guo et al., 2022a)	Direct training	VGG16	LIF	5	70.18%
	Real Spike (Guo et al., 2022c)	Direct training	ResNet20	LIF	5	66.60%
			VGG16		5	70.62%
	Dspike (Li et al., 2021)	Direct training	ResNet20	LIF	2	71.68%
					4	73.35%
	TET (Deng et al., 2022)	Direct training	ResNet18	LIF	2	72.87%
					4	74.47%
	InfLoR-SNN (Guo et al., 2023a)	Direct training	ResNet20	LIF	5	71.19%
			VGG16	LIF	5	71.56%
					10	73.17%
	MPBN (Guo et al., 2023c)	Direct training	VGG16	LIF	4	74.74%
			ResNet20	LIF	2	70.79%
					4	72.30%
	**AdaLi** (ours)	Direct training	VGG16	LIF	4	**74.27%**±0.06
			ResNet18	LIF	2	**75.32%**±0.09
					4	**77.89%**±0.04

Bold values indicate the results of our proposed method (AdaLi).

**Table 3:** Comparison of our method's performance with other approaches on the neuromorphic dataset.

**Dataset**	**Method**	**Type**	**Architecture**	**Neuron model**	**Timestep**	**Accuracy**
DVS-CIFAR-10	SATP (Zhang et al., 2024)	ANN-to-SNN conversion	VGG16	RMP-IF	230	67.09%
	MPD-ATP (Shan et al., 2025)	Direct training	\	LIF	20	74.93%
	HP-SNN (Wu et al., 2022)	Hybrid training	\	LIF	50	67.81%
	Rollout (Kugele et al., 2020)	Streaming	DenseNet	IF	10	66.80%
		ANN-to-SNN conversion	DenseNet		10	65.61%
	STBP-tdBN (Zheng et al., 2021)	Direct training	ResNet18	Iterative LIF	10	67.80%
	RSNN (Xu Q. et al., 2024)	Direct training	RSNN	LIF	\	67.10%
	AdaLi (ours)	Direct training	VGG11	IF	16	**74.7%**±0.04
			VGG16		16	**73.7%**±0.06
DVSGesture	STBP (He et al., 2020)	Direct training	CNN-based	LIF	25	93.40%
	MPD-ATP (Shan et al., 2025)	Direct training	\	LIF	20	97.35%
	HP-SNN (Wu et al., 2022)	Hybrid training	\	LIF	400	97.01%
	MISNN (Liu et al., 2021)	Motion information	CNN-based	LIF	\	92.7%
	SATP (Zhang et al., 2024)	ANN-to-SNN conversion	SCRNN	LIF	389	82.59%
	RSNN (Xu Q. et al., 2024)	Direct training	RSNN	LIF	\	95.10%
	AdaLi (ours)	Direct training	DVSGestureNet	IF	16	**96.87%**±0.02
			VGG11	IF	16	**94.79%**±0.07

Bold values indicate the results of our proposed method (AdaLi).

For neuromorphic datasets, AdaLi achieved a top classification accuracy of 68.4% on DVS-CIFAR-10 using ResNet18 with 10 time steps, outperforming (Zheng et al., 2021) by 0.6% at the same time steps. On the DVSGesture dataset, AdaLi attained an accuracy of 96.87% with 16 time steps on DVSGestureNet, demonstrating superior performance with lower latency compared to (Zhang et al. 2024) and (He et al. 2020).

### 4.2 Hyperparameters selection

Determining the optimal threshold *V*_*th*_ for membrane potential gradients that require updates is critical aspect for ensuring training stability and efficiency. Incorrect gradient boundaries, whether too high or too low, can lead to unstable training dynamics. Furthermore, excessively broad gradient boundaries increase the computational burden during backpropagation, counteracting the energy-efficient potential of SNNs.

**Figure 4:**
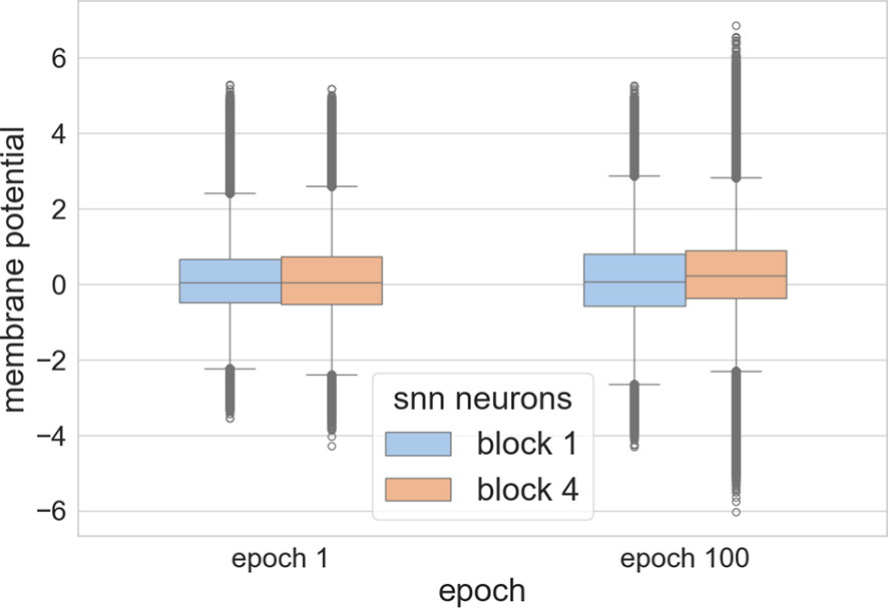
The membrane potential distributions of spiking neurons in the first and fourth blocks of ResNet18 at epoch 1 and 100 during training on CIFAR-10 without adaptive function.

In our experiments with a ResNet18 architecture trained on the CIFAR-10 dataset without an adaptive mechanism, we mapped the membrane potential distributions across various layers at different training epochs. As shown in Figure 4, outliers in the neuronal membrane potential data was observed. We initially set the boundary value to approximately 1.5 times the threshold *V*_*th*_, allowing for the calculation of gradients in over half of the neuronal membrane potentials, thereby promoting parameter updates for a large fraction of the network. We set p = 0.2 in Equation 19. According to statistics, only about 20% of the parameter gradients are computed and updated.

This adaptive function approach balances the training regimen effectively. It maintains stability during the early stages of training and gradually reduces computational intensity as the training progresses. Additionally, under the same experimental conditions but with the adaptive function set to a logarithmic rate of change, we investigated how different membrane potential boundaries requiring gradient updates and gradient intensity impact factors affected network performance on the CIFAR-10 dataset.

**Figure 5:**
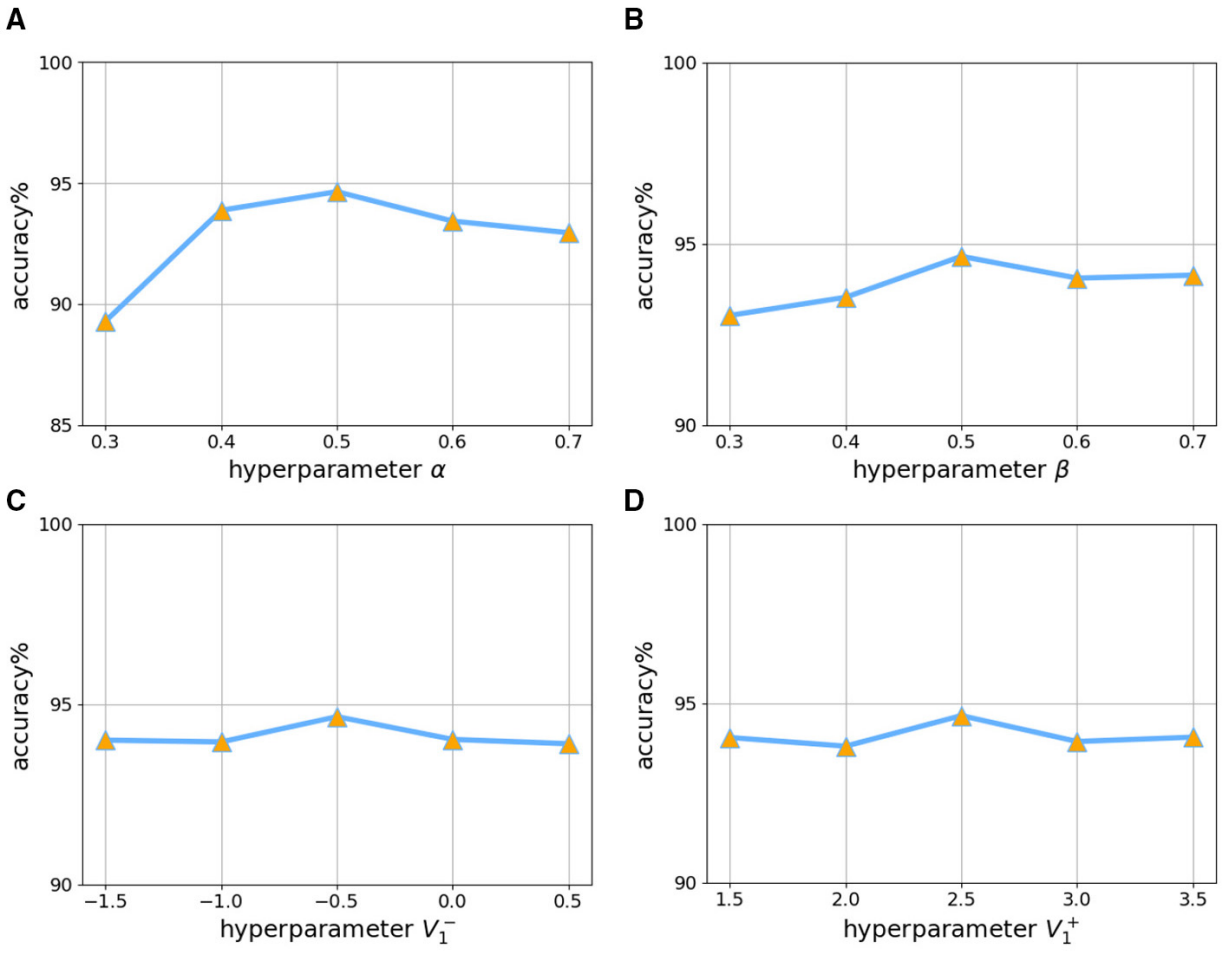
The impact of hyperparameter selection on the performance of training ResNet18 on the CIFAR-10 dataset when the threshold value is equal to 1. **(a)** β = 0.5, $V_{1}^{-}=-0.5$, $V_{1}^{+}=2.5$. **(b)** α = 0.5, $V_{1}^{-}=-0.5$, $V_{1}^{+}=2.5$. **(c)** α = 0.5, β = 0.5, $V_{1}^{-}=-0.5$. **(d)** α = 0.5, β = 0.5, $V_{1}^{+}=2.5$.

The results, as shown in Figure 5, indicate that network performance is highly sensitive to changes in the gradient intensity impact factor. An optimal value for this factor was identified around 0.5; deviations from this median, either higher or lower, significantly degraded network performance. Conversely, network performance shows comparatively less sensitivity to variations in the membrane potential boundary for gradient updates, remaining relatively stable within a certain range. Our findings suggest that setting the boundary value to approximately 1.5 times the threshold value is judicious.

Although these experiments for hyperparameter selection were conducted using the CIFAR-10 dataset, subsequent experiments across different datasets and networks, using the optimal hyperparameters identified here, achieved good performance. This demonstrates the generalization ability of these hyperparameters.

### 4.3 Impact of boundary size

**Figure 6:**
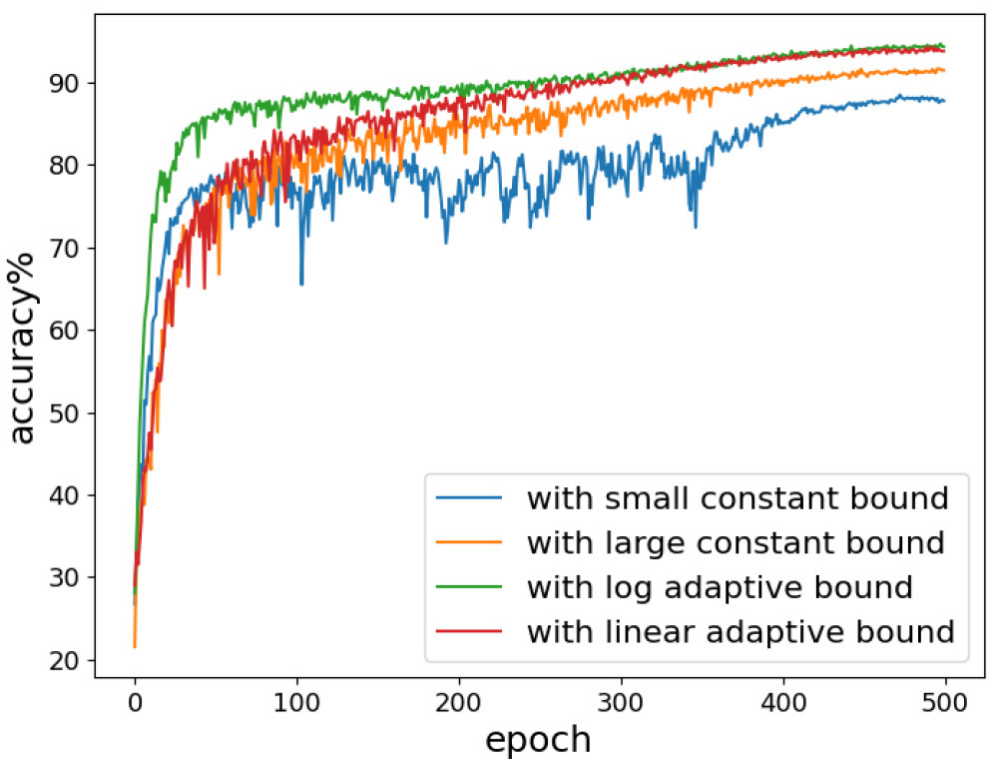
The results of training ResNet18 on the CIFAR-10 dataset using the AdaLi method and only lightweight surrogate gradients. The large constant bound refers to 1.5 times the threshold, which corresponds to the initial value of the adaptive boundary at the beginning of training. The small constant bound refers to the boundary value when *p* = 0.2, which corresponds to the final value of the adaptive boundary at the end of training.

We investigate the impact of different boundary sizes on training outputs. We conducted experiments by training ResNet18 on the CIFAR-10 dataset, setting the time step of spiking neurons to 1. As illustrated in Figure 6, directly setting a small constant boundary without using an adaptive function to adjust the boundary size adaptively results in unstable network training. Significant fluctuation occurs during the mid-term training phase, although it decreases in the later stages.

This observation implies that a larger update boundary is necessary during the early to mid-term phases of training. Conversely, reducing the gradient update boundary in the later stages can help lower computational costs. This behavior aligns with our proposed adaptive function. The orange curve in Figure 6 represents a scenario with a large update boundary. An excessively large update boundary might lead to gradient calculations for membrane potential outliers, resulting in lower accuracy compared to the method employing an adaptive function.

Both adaptive functions, which adjust the boundary size at different rates, eventually achieve high classification accuracy. This demonstrates the efficacy of adaptively adjusting the boundary size throughout the training process.

### 4.4 Firing rate

One major advantage of SNNs is the sparse firing property of spiking neurons. Sparse firing enhances the efficiency of information representation, as only activated neurons fire spikes. This selective firing reduces energy consumption compared to continuously active neurons. The event-driven nature of SNNs allows them to adapt well to neuromorphic hardware, which also relies on event-driven processing. This means that processing is triggered only when there are changes in inputs or specific conditions are met, thereby improving system efficiency and reducing processing complexity.

**Figure 7:**
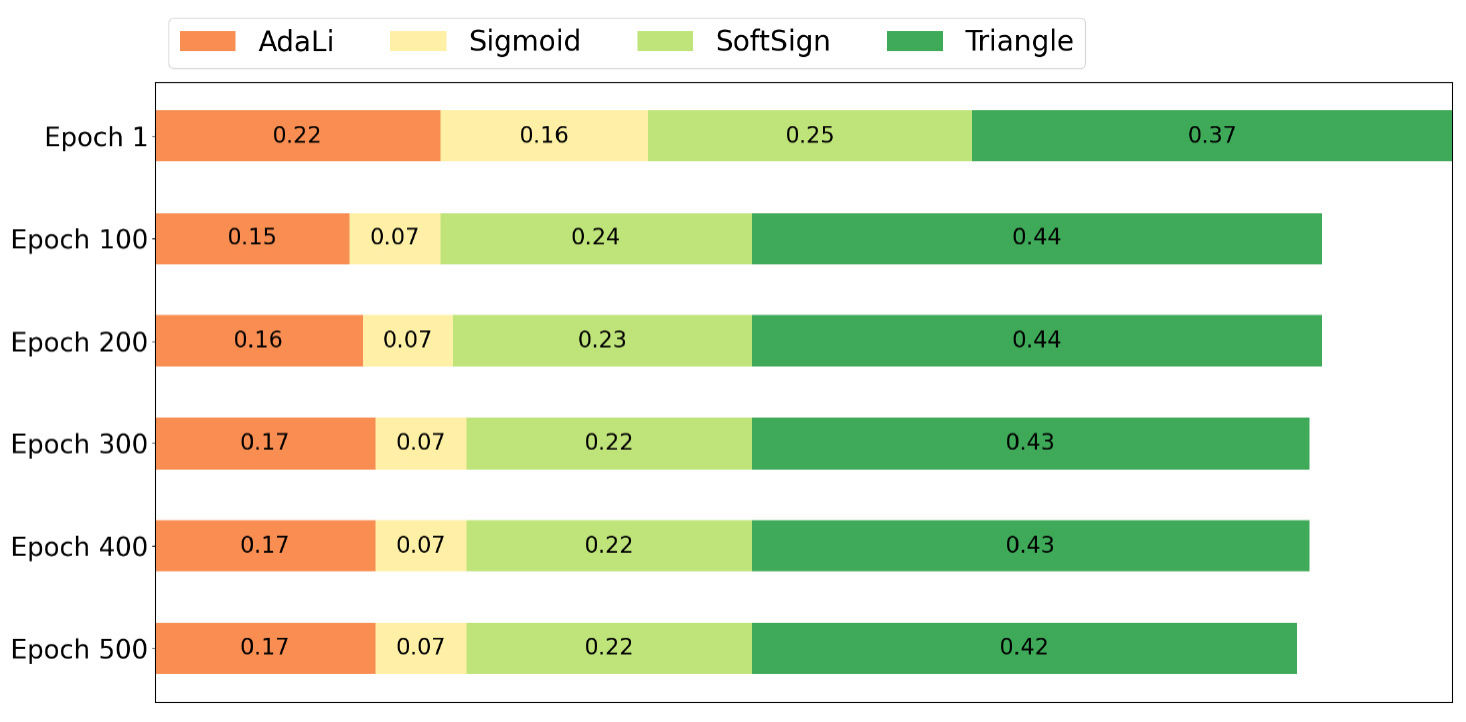
Comparison of the firing rates of different surrogate gradients in SNNs trained on the CIFAR-10 dataset using the ResNet18 model for 500 epochs.

Maintaining the sparse firing property of spiking neurons offers numerous benefits. We recorded the spiking activity of all neurons in the SNN during inference, after training on the CIFAR-10 dataset for 500 epochs using the ResNet18 model and various surrogate gradients. The adaptive function in the AdaLi method is a logarithmic adaptive function, while the parameters for other traditional surrogate gradients remain consistent with those in Table 4. As shown in Figure 7, compared to the SoftSign (Zenke and Ganguli, 2018) and Triangle (Bellec et al., 2018) surrogate gradients, the AdaLi method significantly reduces the spiking rate while maintaining high performance. This balance ensures that sufficient information is transmitted for the network to make accurate predictions, while reducing the spike rate to decrease the computational load during inference.

**Table 4:** Comparison of the performance of different surrogate gradients in SNNs trained on the CIFAR-10 dataset using the ResNet18 model for 500 epochs.

**Setting**	**Accuracy**
Sigmoid, α = 1	69.55%±0.11%
SoftSign, α = 2	83.69%±0.06%
Triangle, α = 1	87.07%±0.05%
AdaLi, α = 0.5, β = 0.5	95.03%±0.07%

Parameters are taken from the optimal case of each method.

Although the Sigmoid (Wu et al., 2018) surrogate gradient shows a much lower spiking rate in Figure 7, Table 4 indicates that using the derivative of the Sigmoid function as the surrogate gradient results in suboptimal training performance. Despite its low spike rate, network performance is negatively affected. Meanwhile, the other two traditional surrogate gradients maintain a relatively higher spiking rate compared to AdaLi. This further demonstrates the superiority of the AdaLi method in achieving high performance with low power consumption.

### 4.5 Computational overheads

We also investigate the impact of AdaLi on computational cost during the training of SNNs. The initial conditions in the experiment are set as detailed in Section 4.3. We record the membrane potential distribution of all spiking neurons in the ResNet18 model and the membrane potential boundary values requiring gradient updates at different training epochs using various surrogate gradients. By calculating the proportion of neurons needing gradient updates, we determine the computational density for each training epoch.

Initially, with the boundary set to 1.5 times the threshold value, about 65% of spiking neurons require gradient updates at the beginning of training. As training progresses, AdaLi reduces the boundary for membrane potential updates, which in turn decreases computational density. Ultimately, only about 20% of spiking neurons need to update gradients. From Figure 6, we observe that even though the log adaptive function has discarded nearly half of the gradient calculations by the 100th epoch, training with this method still outperforms the linear adaptive function. This indicates that the calculation of gradients for most spiking neurons can be omitted without affecting performance.

**Figure 8:**
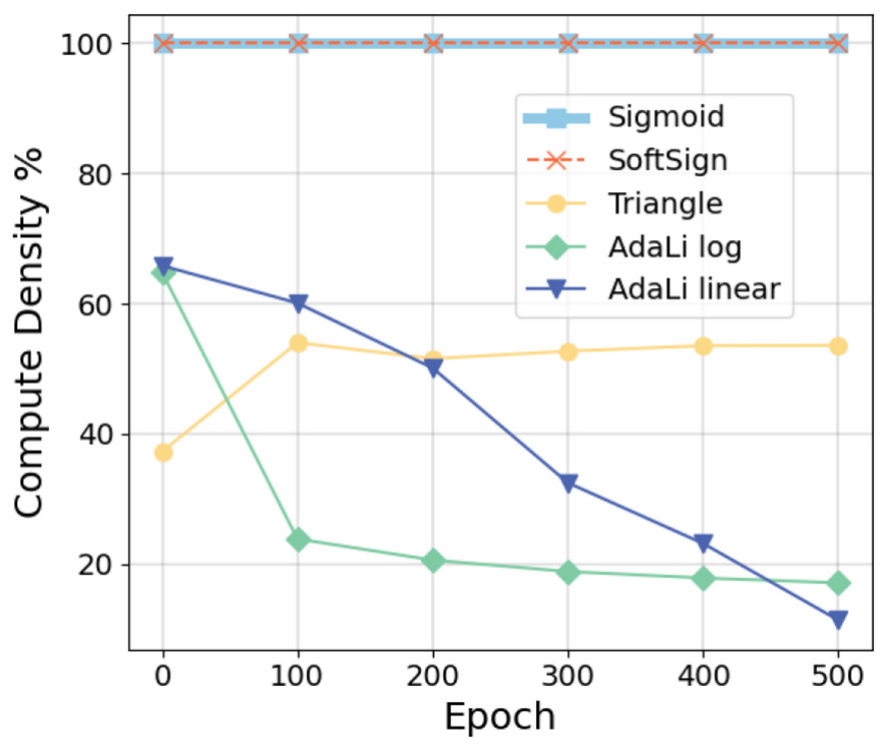
Comparison of the compute density of different surrogate gradients in SNNs trained on the CIFAR-10 dataset using the ResNet18 model for 500 epochs. A computational density of 100% means that the gradient of the membrane potential of each spiking neuron needs to be calculated.

Figure 8 compares the computational density of different surrogate gradient methods of SNNs trained on the CIFAR-10 dataset using the ResNet18 model for 500 epochs. A computational density of 100% means that the gradient of the membrane potential of each spiking neuron needs to be calculated. Our AdaLi method, including the log and linear adaptive functions, shows a continuous reduction in computational density as training progresses, ultimately dropping below 20%. Especially after 100 epochs, AdaLi reduces computational density by approximately 80% compared to the Sigmoid and SoftSign methods, and by about 30% compared to the Triangle method, without compromising network performance. AdaLi's adaptive gradient update mechanism brings a notable boost in actual training efficiency, outperforming other traditional surrogate gradient methods by a large margin in training speed while maintaining high model accuracy.

### 4.6 Feature visualization

**Figure 9:**
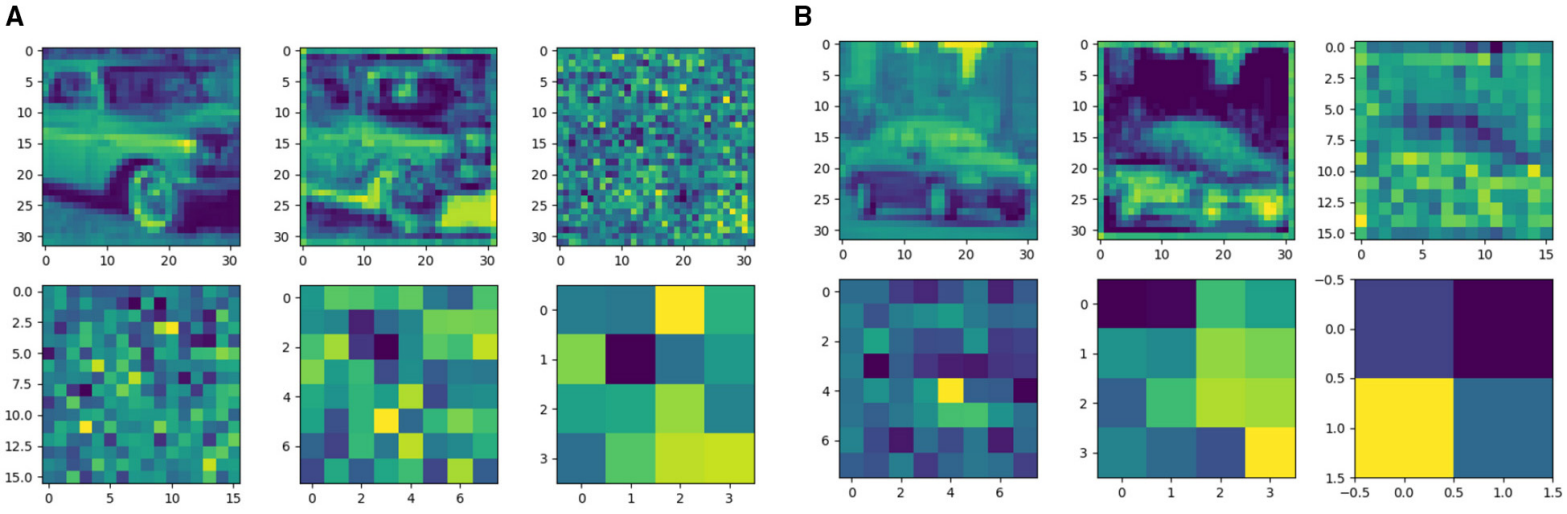
Feature representation of spiking ResNet18 **(a)** and VGG16 **(b)**. The first two subfigures show the feature before and after the coding layer. The last four subfigures show the feature representation after each block.

Figure 9 illustrate the feature representations of ResNet18 and VGG16, respectively. The first two sub-figures depict the network's encoding of input information before and after training. The subsequent four sub-figures show the feature maps after the last convolutional layer of each block extracts the input information. It can be observed that, similar to conventional ANNs, the features in SNN models become more abstract as the network depth increases. Furthermore, different network architectures exhibit varying levels of granularity in feature extraction.

Generally, before entering the classification layer, it is desirable to reduce the spatial dimensions of the feature map while increasing its depth (i.e., the number of channels). Smaller spatial dimensions enable each position in the feature map to capture more localized information, while greater depth allows the capture of richer and more abstract features. Increasing the depth helps the model learn more complex image features, thereby enhancing classification performance.

Notably, after passing through all blocks, our ResNet18 model retains relatively large spatial dimensions in its feature maps, whereas the VGG16 model reduces the spatial dimensions more significantly. Consequently, a pooling layer is employed in ResNet18 before the classification layer to reduce the spatial dimensions and improve classification results.

## 5 Conclusion

This work addresses the challenges of gradient mismatch, gradient vanishing/explosion, and high computational complexity in SNN training. We propose a lightweight gradient to alleviate the computational burden associated with surrogate gradients during backpropagation. The stable nature of these lightweight gradient values helps to mitigate issues related to gradient vanishing and explosion. Additionally, the gradient intensity factor and update boundary in the AdaLi method collaboratively address the gradient mismatch problem. The application of the adaptive function in our approach effectively reduces computational load during backpropagation while maintaining training stability. We also provide optimal initial and final values for the update boundary and verifies the effectiveness of the adaptive function. Leveraging these advancements, AdaLi achieves stable, high-performance training with reduced computational costs.

One limitation of this work is the manual selection of the gradient update range, which can introduce some subjectivity into the process. Additionally, the determination of optimal hyperparameters is based on empirical experimentation rather than theoretical foundations. Future work can aim for a deeper investigation into the relationship between the update range and the threshold, with the goal of developing a more systematic and theoretically grounded method for parameter selection. Another promising direction is exploring the underlying causes of the sensitivity of experimental results to the gradient intensity factor, which could provide a clearer understanding of its impact on model performance. Furthermore, investigating the use of more suitable adaptive functions for dynamically updating the gradient range could enhance model robustness and reduce sensitivity to hyperparameter selection. By addressing these limitations, future research can refine the proposed methods and improve their generalizability and performance across various tasks and datasets.

## Data Availability

The datasets presented in this study can be found in online repositories. The names of the repository/repositories and accession number(s) can be found below: https://github.com/paran-ia/AdaLi.

## References

[B1] Amir A. Taba B. Berg D. Melano T. McKinstry J. Di Nolfo C. . (2017). “A low power, fully event-based gesture recognition system,” in Proceedings of the IEEE conference on Computer Vision and Pattern Recognition (Honolulu, HI: IEEE), 7243–7252.

[B2] Baronig M. Ferrand R. Sabathiel S. Legenstein R. (2025). Advancing spatio-temporal processing through adaptation in spiking neural networks. Nat. Commun. 16:5776. doi: 10.1038/s41467-025-60878-z 40593685 PMC12218304

[B3] Bellec G. Salaj D. Subramoney A. Legenstein R. Maass W. (2018). “Long short-term memory and learning-to-learn in networks of spiking neurons,” in Advances in Neural Information Processing Systems (Red Hook, NY: Curran Associates, Inc.), 31.

[B4] Bewley A. Ge Z. Ott L. Ramos F. Upcroft B. (2016). “Simple online and realtime tracking,” in 2016 IEEE interNATIONAL conference on Image Processing (ICIP) (Phoenix, AZ: IEEE), 3464–3468.

[B5] Bohte S. M. Kok J. N. La Poutre H. (2002). Error-backpropagation in temporally encoded networks of spiking neurons. Neurocomputing 48, 17–37. doi: 10.1016/S0925-2312(01)00658-0

[B6] Brzosko Z. Mierau S. B. Paulsen O. (2019). Neuromodulation of spike-timing-dependent plasticity: Past, present, and future. Neuron 103, 563–581. doi: 10.1016/j.neuron.2019.05.041 31437453

[B7] Burkitt A. N. (2006). A review of the integrate-and-fire neuron model: I. homogeneous synaptic input. Biol. Cybernet. 95, 1–19. doi: 10.1007/s00422-006-0068-6 16622699

[B8] Cai Z. Kalatehbali H. R. Walters B. Rahimi Azghadi M. Amirsoleimani A. Genov R. (2023). Spike timing dependent gradient for direct training of fast and efficient binarized spiking neural networks. IEEE J. Emerg. Select. Topics Circuits Syst. 13, 1083–1093. doi: 10.1109/JETCAS.2023.3328926

[B9] Caporale N. Dan Y. (2008). Spike timing-dependent plasticity: a hebbian learning rule. Annu. Rev. Neurosci. 31, 25–46. doi: 10.1146/annurev.neuro.31.060407.125639 18275283

[B10] Cheng X. Hao Y. Xu J. Xu B. (2020). “LISNN: Improving spiking neural networks with lateral interactions for robust object recognition,” in Proceedings of the Twenty-Ninth International Joint Conference on Artificial Intelligence (IJCAI) (Palo Alto, CA: AAAI Press/ijcai.org), 1519–1525.

[B11] Dan Y. Poo M. (2004). Spike timing-dependent plasticity of neural circuits. Neuron 44, 23–30. doi: 10.1016/j.neuron.2004.09.007 15450157

[B12] Davies M. Srinivasa N. Lin T.-H. Chinya G. Cao Y. Choday S. H. . (2018). Loihi: A neuromorphic manycore processor with on-chip learning. IEEE Micro 38, 82–99. doi: 10.1109/MM.2018.112130359

[B13] Debanne D. Inglebert Y. (2023). Spike timing-dependent plasticity and memory. Curr. Opini. Neurobiol. 80:102707. doi: 10.1016/j.conb.2023.102707 36924615

[B14] Deng S. Li Y. Zhang S. Gu S. (2022). Temporal efficient training of spiking neural network via gradient re-weighting. arXiv [preprint] arXiv:2202.11946. doi: 10.48550/arXiv.2202.11946

[B15] Diehl P. U. Cook M. (2015). Unsupervised learning of digit recognition using spike-timing-dependent plasticity. Front. Comput. Neurosci. 9:99. doi: 10.3389/fncom.2015.00099 26941637 PMC4522567

[B16] Eshraghian J. K. Ward M. Neftci E. O. Wang X. Lenz G. Dwivedi G. . (2023). “Training spiking neural networks using lessons from deep learning,” in Proceedings of the IEEE (Piscataway, NJ: IEEE).

[B17] Fang W. Yu Z. Chen Y. Huang T. Masquelier T. Tian Y. (2021a). “Deep residual learning in spiking neural networks,” in Advances in Neural Information Processing Systems (Red Hook, NY: Curran Associates, Inc.), 34, 21056–21069.

[B18] Fang W. Yu Z. Chen Y. Masquelier T. Huang T. Tian Y. (2021b). “Incorporating learnable membrane time constant to enhance learning of spiking neural networks,” in Proceedings of the IEEE/CVF International Conference on Computer Vision (Montreal, QC: IEEE), 2661–2671.

[B19] Guo Y. Chen Y. Liu X. Peng W. Zhang Y. Huang X. . (2024). “Ternary spike: Learning ternary spikes for spiking neural networks,” in Proceedings of the AAAI Conference on Artificial Intelligence (Washington, DC: AAAI Press), 38, 12244–12252.

[B20] Guo Y. Chen Y. Zhang L. Liu X. Wang Y. Huang X. . (2022a). “IM-loss: information maximization loss for spiking neural networks,” in Advances in Neural Information Processing Systems (Red Hook, NY: Curran Associates, Inc.) 35, 156–166.

[B21] Guo Y. Chen Y. Zhang L. Wang Y. Liu X. Tong X. . (2023a). “InfLoR-SNN: reducing information loss for spiking neural networks,” in Computer Vision-ECCV 2022, 36–52.

[B22] Guo Y. Huang X. Ma Z. (2023b). Direct learning-based deep spiking neural networks: a review. Front. Neurosci. 17:1209795. doi: 10.3389/fnins.2023.1209795 37397460 PMC10313197

[B23] Guo Y. Tong X. Chen Y. Zhang L. Liu X. Ma Z. . (2022b). “RecDis-SNN: Rectifying membrane potential distribution for directly training spiking neural networks,” in Proceedings of the IEEE/CVF Conference on Computer Vision and Pattern Recognition (New Orleans, LA: IEEE), 326–335.

[B24] Guo Y. Zhang L. Chen Y. Tong X. Liu X. Wang Y. . (2022c). “Real spike: Learning real-valued spikes for spiking neural networks,” in European Conference on Computer Vision (Cham: Springer), 52–68.

[B25] Guo Y. Zhang Y. Chen Y. Peng W. Liu X. Zhang L. . (2023c). Membrane potential batch normalization for spiking neural networks. arXiv [preprint] arXiv:2308.08359. doi: 10.1109/ICCV51070.2023.01779

[B26] He K. Zhang X. Ren S. Sun J. (2016). “Deep residual learning for image recognition,” in Proceedings of the IEEE Conference on Computer Vision and Pattern Recognition (Las Vegas, NV: IEEE), 770–778.

[B27] He W. Wu Y. Deng L. Li G. Wang H. Tian Y. . (2020). Comparing SNNs and RNNs on neuromorphic vision datasets: similarities and differences. Neural Netw. 132, 108–120. doi: 10.1016/j.neunet.2020.08.001 32866745

[B28] Histed M. H. Bonin V. Reid R. C. (2009). Direct activation of sparse, distributed populations of cortical neurons by electrical microstimulation. Neuron 63, 508–522. doi: 10.1016/j.neuron.2009.07.016 19709632 PMC2874753

[B29] Hong C. Wei X. Wang J. Deng B. Yu H. Che Y. (2019). Training spiking neural networks for cognitive tasks: a versatile framework compatible with various temporal codes. IEEE trans. Neural Netw. Learn. Syst. 31, 1285–1296. doi: 10.1109/TNNLS.2019.2919662 31247574

[B30] Krizhevsky A. Hinton G. (2009). “Learning multiple layers of features from tiny images,” in Handbook of Systemic Autoimmune Diseases (Toronto, ON: University of Toronto), 1.

[B31] Kugele A. Pfeil T. Pfeiffer M. Chicca E. (2020). Efficient processing of spatio-temporal data streams with spiking neural networks. Front. Neurosci. 14:439. doi: 10.3389/fnins.2020.00439 32431592 PMC7214871

[B32] Li H. Liu H. Ji X. Li G. Shi L. (2017). Cifar10-dvs: an event-stream dataset for object classification. Front. Neurosci. 11:309. doi: 10.3389/fnins.2017.00309 28611582 PMC5447775

[B33] Li Y. Guo Y. Zhang S. Deng S. Hai Y. Gu S. (2021). “Differentiable spike: Rethinking gradient-descent for training spiking neural networks,” in Advances in Neural Information Processing Systems (Red Hook, NY: Curran Associates, Inc.), 34, 23426–23439.

[B34] Lian S. Shen J. Wang Z. Tang H. (2024). IM-LIF: improved neuronal dynamics with attention mechanism for direct training deep spiking neural network. IEEE Trans. Emerg. Topics Comput. Intellig. 8, 2075–2085. doi: 10.1109/TETCI.2024.3359539

[B35] Liang L. Hu X. Deng L. Wu Y. Li G. Ding Y. . (2023). Exploring adversarial attack in spiking neural networks with spike-compatible gradient. IEEE Trans. Neural Netw. Learn. Syst. 34, 2569–2583. doi: 10.1109/TNNLS.2021.3106961 34473634

[B36] Liu L. Prost J. Zhu L. Papadakis N. Liò P. Schönlieb C.-B. . (2023). “Scotch and soda: A transformer video shadow detection framework,” in Proceedings of the IEEE/CVF Conference on Computer Vision and Pattern Recognition (Vancouver, BC: IEEE), 10449–10458.

[B37] Liu Q. Xing D. Tang H. Ma D. Pan G. (2021). “Event-based action recognition using motion information and spiking neural networks,” in Proceedings of the Thirtieth International Joint Conference on Artificial Intelligence (IJCAI), 1743–1749.

[B38] Liu Y. Chen Z. Zhao W. Zhao T. Jia T. Wang Z. . (2024). Sparsity-aware in-memory neuromorphic computing unit with configurable topology of hybrid spiking and artificial neural network. IEEE Trans. Circuits and Syst. I: Regular Papers 71, 2660–2673. doi: 10.1109/TCSI.2024.3377700

[B39] Luo X. Qu H. Wang Y. Yi Z. Zhang J. Zhang M. (2023). Supervised learning in multilayer spiking neural networks with spike temporal error backpropagation. IEEE Trans. Neural Netw. Learn. Syst. 34:10141–10153. doi: 10.1109/TNNLS.2022.3164930 35436200

[B40] Mainen Z. F. Sejnowski T. J. (1995). Reliability of spike timing in neocortical neurons. Science 268, 1503–1506. doi: 10.1126/science.7770778 7770778

[B41] Masquelier T. Thorpe S. J. (2007). Unsupervised learning of visual features through spike timing dependent plasticity. PLoS Comput. Biol. 3:e31. doi: 10.1371/journal.pcbi.0030031 17305422 PMC1797822

[B42] Meng Q. Xiao M. Yan S. Wang Y. Lin Z. Luo Z.-Q. (2022). “Training high-performance low-latency spiking neural networks by differentiation on spike representation,” in Proceedings of the IEEE/CVF Conference on Computer Vision and Pattern Recognition (New Orleans, LA: IEEE), 12444–12453.

[B43] Meng Q. Xiao M. Yan S. Wang Y. Lin Z. Luo Z.-Q. (2023). Towards memory-and time-efficient backpropagation for training spiking neural networks. arXiv [preprint] arXiv:2302.14311. doi: 10.1109/ICCV51070.2023.00567

[B44] Merolla P. A. Arthur J. V. Alvarez-Icaza R. Cassidy A. S. Sawada J. Akopyan F. . (2014). A million spiking-neuron integrated circuit with a scalable communication network and interface. Science 345, 668–673. doi: 10.1126/science.1254642 25104385

[B45] Mostafa H. (2018). Supervised learning based on temporal coding in spiking neural networks. IEEE Trans. Neural Netw. Learn. Syst. 29, 3227–3235. doi: 10.1109/TNNLS.2018.2814526 28783639

[B46] Neftci E. O. Mostafa H. Zenke F. (2019). Surrogate gradient learning in spiking neural networks: Bringing the power of gradient-based optimization to spiking neural networks. IEEE Signal Proc. Magazine 36, 51–63. doi: 10.1109/MSP.2019.2931595

[B47] Painkras E. Plana L. A. Garside J. Temple S. Galluppi F. Patterson C. . (2013). Spinnaker: A 1-w 18-core system-on-chip for massively-parallel neural network simulation. IEEE J. Solid-State Circuits 48, 1943–1953. doi: 10.1109/JSSC.2013.2259038

[B48] Pei J. Deng L. Song S. Zhao M. Zhang Y. Wu S. . (2019). Towards artificial general intelligence with hybrid tianjic chip architecture. Nature 572, 106–111. doi: 10.1038/s41586-019-1424-8 31367028

[B49] Rahman N. A. Yusoff N. (2025). Modulated spike-time dependent plasticity (STDP)-based learning for spiking neural network (SNN): A review. Neurocomputing 618:129170. doi: 10.1016/j.neucom.2024.129170

[B50] Rançon U. Cuadrado-Anibarro J. Cottereau B. R. Masquelier T. (2022). Stereospike: Depth learning with a spiking neural network. IEEE Access 10, 127428–127439. doi: 10.1109/ACCESS.2022.3226484

[B51] Rathi N. Roy K. (2020). Diet-SNN: Direct input encoding with leakage and threshold optimization in deep spiking neural networks. arXiv [preprint] arXiv:2008.03658. doi: 10.48550/arXiv.2008.03658 34596559

[B52] Ronneberger O. Fischer P. Brox T. (2015). “U-Net: convolutional networks for biomedical image segmentation,” in Medical Image Computing and Computer-Assisted Intervention-MICCAI 2015: 18th International Conference, Munich, Germany, October 5-9, 2015, Proceedings, Part III 18 (Munich: Springer), 234–241.

[B53] Rueckauer B. Lungu I.-A. Hu Y. Pfeiffer M. Liu S.-C. (2017). Conversion of continuous-valued deep networks to efficient event-driven networks for image classification. Front. Neurosci. 11:682. doi: 10.3389/fnins.2017.00682 29375284 PMC5770641

[B54] Saponati M. Vinck M. (2023). Sequence anticipation and spike-timing-dependent plasticity emerge from a predictive learning rule. Nat. Commun. 14:4985. doi: 10.1038/s41467-023-40651-w 37604825 PMC10442404

[B55] Shan N. Lin Y. Xie C. Wu K. Ma W. Mu C. . (2025). Membrane potential-driven adaptive threshold plasticity for SNNs: A bio-inspired mechanism combining inverse depolarization rate and proportional membrane potential dynamics. IEEE Trans. Emerg. Topics Comput. Intellig. 2025, 1–13. doi: 10.1109/TETCI.2025.3631624

[B56] Shen H. Zheng Q. Wang H. Pan G. (2024). “Rethinking the membrane dynamics and optimization objectives of spiking neural networks,” in Advances in Neural Information Processing Systems (Red Hook, NY: Curran Associates, Inc.), 37, 92697–92720.

[B57] Simonyan K. Zisserman A. (2014). Very deep convolutional networks for large-scale image recognition. arXiv [preprint] arXiv:1409.1556. doi: 10.48550/arXiv.1409.1556

[B58] Stanojevic A. Woźniak S. Bellec G. Cherubini G. Pantazi A. Gerstner W. (2024). High-performance deep spiking neural networks with 0.3 spikes per neuron. Nature Commun. 15:6793. doi: 10.1038/s41467-024-51110-5 39122775 PMC11315905

[B59] Su Q. Chou Y. Hu Y. Li J. Mei S. Zhang Z. . (2023). “Deep directly-trained spiking neural networks for object detection,” in 2023 IEEE/CVF International Conference on Computer Vision (ICCV) (Piscataway, NJ: IEEE), 6532–6542.

[B60] Tavanaei A. Maida A. S. (2016). Bio-inspired spiking convolutional neural network using layer-wise sparse coding and stdp learning. arXiv [preprint] arXiv:1611.03000. doi: 10.48550/arXiv.1611.03000

[B61] Wang C.-Y. Bochkovskiy A. Liao H.-Y. M. (2023). “YOLOv7: Trainable bag-of-freebies sets new state-of-the-art for real-time object detectors,” in Proceedings of the IEEE/CVF Conference on Computer Vision and Pattern Recognition (Vancouver, BC: IEEE), 7464–7475.

[B62] Wei W. Zhang M. Zhang J. Belatreche A. Wu J. Xu Z. . (2024). Event-driven learning for spiking neural networks. arXiv [preprint] arXiv:2403.00270. doi: 10.48550/arXiv.2403.00270

[B63] Wu Y. Deng L. Li G. Zhu J. Shi L. (2018). Spatio-temporal backpropagation for training high-performance spiking neural networks. Front. Neurosci. 12:331. doi: 10.3389/fnins.2018.00331 29875621 PMC5974215

[B64] Wu Y. Deng L. Li G. Zhu J. Xie Y. Shi L. (2019). “Direct training for spiking neural networks: faster, larger, better,” in Proceedings of the Thirty-Third AAAI Conference on Artificial Intelligence and Thirty-First Innovative Applications of Artificial Intelligence Conference and Ninth AAAI Symposium on Educational Advances in Artificial Intelligence, AAAI'19/IAAI'19/EAAI'19 (Washington, DC: AAAI Press).

[B65] Wu Y. Zhao R. Zhu J. Chen F. Xu M. Li G. . (2022). Brain-inspired global-local learning incorporated with neuromorphic computing. Nat. Commun. 13:65. doi: 10.1038/s41467-021-27653-2 35013198 PMC8748814

[B66] Xiao M. Meng Q. Zhang Z. He D. Lin Z. (2022). “Online training through time for spiking neural networks,” in Advances in Neural Information Processing Systems, eds. S. Koyejo, S. Mohamed, A. Agarwal, D. Belgrave, K. Cho, and A. Oh (Red Hook, NY: Curran Associates, Inc.), 20717–20730.

[B67] Xu Q. Fang X. Li Y. Shen J. Ma D. Xu Y. . (2024). “RSNN: Recurrent spiking neural networks for dynamic spatial-temporal information processing,” in ACM Multimedia Conference (New York, NY: Association for Computing Machinery (ACM)).

[B68] Xu Z. You K. Guo Q. Wang X. He Z. (2024). BKDSNN: Enhancing the performance of learning-based spiking neural networks training with blurred knowledge distillation. arXiv [preprint] arXiv:2407.09083. doi: 10.1007/978-3-031-72973-7_7

[B69] Yamazaki K. Vo-Ho V.-K. Bulsara D. Le N. (2022). Spiking neural networks and their applications: a review. Brain Sci. 12:863. doi: 10.3390/brainsci12070863 35884670 PMC9313413

[B70] Yang Q. Wu J. Zhang M. Chua Y. Wang X. Li H. (2022). “Training spiking neural networks with local tandem learning,” in Advances in Neural Information Processing Systems (Red Hook, NY: Curran Associates, Inc.) 35, 12662–12676.

[B71] Yao M. Zhao G. Zhang H. Hu Y. Deng L. Tian Y. . (2023). Attention spiking neural networks. IEEE Trans. Pattern Analy. Mach. Intellig. 45, 9393–9410. doi: 10.1109/TPAMI.2023.3241201 37022261

[B72] Ye W. Chen Y. Liu Y. (2023). The implementation and optimization of neuromorphic hardware for supporting spiking neural networks with mlp and cnn topologies. IEEE Trans. Comp.-Aided Design Integrat. Circuits Syst. 42, 448–461. doi: 10.1109/TCAD.2022.3179246

[B73] Zenke F. Ganguli S. (2018). Superspike: Supervised learning in multilayer spiking neural networks. Neural Comp. 30, 1514–1541. doi: 10.1162/neco_a_01086 29652587 PMC6118408

[B74] ZenkeF. VogelsT. P. (2021). The remarkable robustness of surrogate gradient learning for instilling complex function in spiking neural networks. Neural Comp. 33, 899–925. doi: 10.1162/neco_a_0136733513328

[B75] Zhang A. Gao Y. Niu Y. Li X. Chen Q. (2023). Intrinsic plasticity for online unsupervised learning based on soft-reset spiking neuron model. IEEE Trans. Cognit. Dev. Syst. 15, 337–347. doi: 10.1109/TCDS.2020.3041610

[B76] Zhang A. Han Y. Niu Y. Gao Y. Chen Z. Zhao K. (2022a). Self-evolutionary neuron model for fast-response spiking neural networks. IEEE Trans. Cognit. Dev. Syst. 14, 1766–1777. doi: 10.1109/TCDS.2021.3139444

[B77] Zhang A. Li X. Gao Y. Niu Y. (2022b). Event-driven intrinsic plasticity for spiking convolutional neural networks. IEEE Trans. Neural Netw. Learn. Syst. 33, 1986–1995. doi: 10.1109/TNNLS.2021.3084955 34106868

[B78] Zhang A. Shi J. Wu J. Zhou Y. Yu W. (2024). Low latency and sparse computing spiking neural networks with self-driven adaptive threshold plasticity. IEEE Trans. Neural Netw. Learn. Syst. 35, 17177–17188. doi: 10.1109/TNNLS.2023.3300514 37581976

[B79] Zhang S. Zhang A. (2025). “Spiking-Siamese-uNet with multi-threshold spiking neurons for post-disaster building damage assessment,” in 2025 Joint International Conference on Automation-Intelligence-Safety (ICAIS) & *International Symposium on Autonomous Systems (ISAS)* (Xi'an: IEEE), 1–7.

[B80] Zhang W. Li P. (2020). “Temporal spike sequence learning via backpropagation for deep spiking neural networks,” in Advances in Neural Information Processing Systems (Red Hook, NY: Curran Associates, Inc.) 33, 12022–12033.

[B81] Zheng H. Wu Y. Deng L. Hu Y. Li G. (2021). “Going deeper with directly-trained larger spiking neural networks,” in Proceedings of the AAAI Conference on Artificial Intelligence, volume 35 (Washington, DC: AAAI Press), 11062–11070.

[B82] Zhou C. Zhang H. Yu L. Ye Y. Zhou Z. Huang L. . (2024). Direct training high-performance deep spiking neural networks: a review of theories and methods. Front. Neurosci. 18:1383844. doi: 10.3389/fnins.2024.1383844 39145295 PMC11322636

[B83] Zhu Y. Yu Z. Fang W. Xie X. Huang T. Masquelier T. (2022). “Training spiking neural networks with event-driven backpropagation,” in Advances in Neural Information Processing Systems 35, 30528–30541.

